# Correspondence

**Published:** 1897-05

**Authors:** Lawrence Campbell

**Affiliations:** Secretary Chicago Veterinary Society; Chicago


### CORRESPONDENCE.

To the Boss Horseshoers' Association of Chicago, Illinois.

Gentlemen: As a body of tradesmen you did all in your power to procure for your craft legislation at the last session of the Legislature at Springfield. No doubt you considered well the advantages this legislation would be to owners of stock if such a bill had passed in the favor of your tradesmen; certainly if such a bill had become a law the public were assured that their horses were being shod under the supervision of tradesmen who were qualified, and knew what shoe was required and if the foot was in a condition to be shod. We are aware that many horseshoeing shops in this city have been run by parties who know nothing about the trade they have entered into, and some parties running around among owners of stock contracting and offering to do work much cheaper than your capable boss horseshoers would think of doing it for, causing you no end of annoyance and loss of customers to many of you, who had earned your knowledge of the business by your many years of apprenticeship and experience under other employers previous to entering into business as a capable and trusty boss horseshoer. Certainly, you gentlemen of the Association thought well of the necessity of the passage of such a bill and what the advantages to the public would be if such would become a law. Gentlemen, in our opinion, your ideas of such a bill were grand. As an Association you had fully realized the necessity of the same, owing to the many parties just referred to starting up places of business and hanging up signs over doors: "John So and So, practical horseshoer." You all considered that such a proceeding was an imposition upon the owners of stock, and you considered a man who was not capable of shoeing a horse ought not to be allowed to represent himself to the public as a boss horseshoer; therefore, the passage of such a bill was necessary for the protection of those who were owners of horses from those unscrupulous men who dared to represent they were capable of doing what they were not. But, again, how many of this Association, who were so very anxious for the protection of the public against such men, state and represent to the public and owners of horses that they are not only boss horseshoers but veterinary surgeons, and capable of attending to all the ills the horse is heir to? As an intelligent body of tradesmen who lately, through your Association, asked for legislation, have you ever considered that those of your Association who, by card and other ways, so wrongfully represent themselves to the public as veterinary surgeons, ought not first to take the mote out of their own eyes? Certainly, gentlemen, your motto is: "Do unto others as we would have others do unto us."

A few practical examples regarding horseshoers acting as veterinary surgeons will call to your mind the fact that we are right in protesting against its continuance. Take "nail wounds," for instance. How many horseshoers are there who will refuse to treat them, telling the owner of the horse that it is the work of the veterinary surgeon to treat it? Do you do this? No. You tell the owner you can fix that up in a day or two. Your treatment (?):

You pour an acid into the wound. You cure a good many cases (?): Yes, if the hole has been so small that the acid did not penetrate into the delicate tissues the horse gets well quickly, provided the germ of lockjaw did not enter with the nail. On the other hand, if the hole was large and the acid enters freely, the horse stays lame until the owner gets disgusted and calls in a veterinary surgeon, who finds one of several conditions—*i. e.*, he enlarges the opening and finds a lot of dead tissue, due to the caustic action of the acid, acting as a foreign body in the foot, keeping up inflammation and pain; frequently upon removal of this, with a day or two of proper treatment, the animal goes sound. Another and more common condition we find is pus being discharged from around the coronet, due to the kind horseshoer stopping up the nail-hole either by the use of the acid, which swells the tissues, or from the aforesaid dead tissue acting as a plug, which prevents the free discharge of pus from the bottom of the hoof. Consequently it works upward, and either greatly prolongs the misery and uselessness of the horse or results in his destruction entirely.

Gentlemen, we will ask you, up to a few months ago, until the founding of your worthy school, how many horseshoers knew how many bones there were in a horse's foot or anything whatever about the blood- and nerve-supply? What do you know about germs--such microscopic germs as cause tetanus (lockjaw), blood-poisoning, and numerous other diseases? What do you know about antiseptics or germ-destroyers? About, say, the pathology of even a nail-wound?

Would *you*, if you stepped on to a nail, go to the man that made your shoes to treat it? We find the same horseshoers who cannot tell us how many bones there are in a horse's foot frequently, yes, daily, dispute with us regarding the location of lameness, usually locating it in the shoulder, the most powerful part of the leg and most free from sprains, overlooking entirely the tender part, especially below the knee and more especially the foot; overlooking entirely the fact that the slender tendons below the knee or complicated joints are more prone to a sprain than the powerful muscles of the shoulder.

Again, take the foot. Few can understand how lameness can exist in it when it is apparently a perfect foot. Yet it can, to external appearances, be a perfect model of a hoof and still be the seat of the malicious navicular disease, or, possibly from an old nail-prick, there is a bulging of horn or perhaps a small growth of bone impinging upon the sensitive structure of the hoof, etc.

Aside from nearly all who make it a point to treat nail-wounds and lameness, there is the horseshoer who is so elated over his smartness that he gets cards printed: "John Jones, veterinary surgeon;" and perhaps he buys a float, and then he is a veterinary surgeon and dentist. What are his qualifications? He has rubbed up against horses a number of years and is the proud possessor of perhaps Kendall's *Spavin Cure, Horse-Doctor Book*, or some similar quack-book.

What does he know? Does he know anything even about the first principles of practice; that is, the pulse and temperature? For instance, a member of this Society a few weeks ago was called to a sick horse. The first thing done was to feel the animal's pulse; from its condition the owner was informed that the horse would die inside of twenty or thirty minutes, when the horseshoer veterinary surgeon stepped up and said: "Doctor, that's also what I think." Upon hearing this the owner spoke up, saying to said quack: "What the d——l do you mean? Only five minutes ago you told me the horse was getting better and could go to work to-morrow." Our member then found that the said horseshoer veterinary surgeon had been treating the animal for a disease he did not have and giving medicine that, under the circumstances, acted as a deadly poison to the horse.

The men of this class know nothing regarding the intricate action of drugs, of their indications or contraindications in disease. They only know about five diseases and about so many prescriptions copied out of a book written by a man equally as ignorant, for the reason that the man who is content with such knowdelge is too ignorant to comprehend the meaning of the language in first-class veterinary works, and yet the woods are full of horseshoers who usurp the duties of the veterinary surgeon. Such men are an injury to yourselves, a detriment to the uplifting of your calling, an injury to the horse, and an enemy to the public. As long as such men exist, so long will your calling be relegated to the ranks of the ignorant. It is your duty to shoe a horse, know when and how to shoe him properly, and correct such faults of the locomotory apparatus as can be corrected by shoeing, and leave the doctoring to those men who are qualified and have studied specially to do so; men who are graduates of recognized veterinary colleges and understand every department of the horse's body, together with its ills and pains, as well as proper treatment.

Gentlemen, we ask you to consider the foregoing statement carefully. Consider it as a friendly argument, coming from your friends, and not from enemies. We are your friends, for how often do we find an animal lame from a nail-prick while being shod; and we invariably, where the owner does not know otherwise, cover the error by not mentioning the real cause, or, where he does know, we help you out and save the customer for you by explaining that such occurrences are due to the nail bending on account of being a little softer or thinner at one part than it should be, or striking an extra hard spot in the wall or a piece of a nail which has remained imbedded in the wall. What return do we get for this kindness?

Gentlemen, we will close by asking you to let doctoring alone. Live and let live. Wishing you success in getting your bill on legislation passed, we remain,

Yours respectfully,

LAWRENCE CAMPBELL, D.V.S.,
Secretary Chicago Veterinary Society.

CHICAGO, Feb. 12, 1897.

